# Bioinformatics-based discovery of PYGM and TNNC2 as potential biomarkers of head and neck squamous cell carcinoma

**DOI:** 10.1042/BSR20191612

**Published:** 2019-07-29

**Authors:** Yu Jin, Ya Yang

**Affiliations:** 1 Department of General Dentistry, Ninth People’s Hospital, Shanghai Jiao Tong University School of Medicine, Shanghai 200011, P.R. China; 2 Shanghai Key Laboratory of Stomatology and Shanghai Research Institute of Stomatology, National Clinical Research Center of Stomatology, 200000, P.R. China

**Keywords:** bioinformatics, biomarker, head and neck squamous cell carcinoma, prognosis, PYGM, TNNC2

## Abstract

Head and neck squamous cell carcinoma (HNSCC) is an aggressive malignancy with high morbidity and mortality rates and ranks as the sixth most common cancer all over the world. Despite numerous advancements in therapeutic methods, the prognosis of HNSCC patients still remains poor. Therefore, there is an urgent need to have a better understanding of the molecular mechanisms underlying HNSCC progression and to identify essential genes that could serve as effective biomarkers and potential treatment targets. In the present study, original data of three independent datasets were downloaded from the Gene Expression Omnibus database (GEO) and R language was applied to screen out the differentially expressed genes (DEGs). PYGM and TNNC2 were finally selected from the overlapping DEGs of three datasets for further analyses. Transcriptional and survival data related to PYGM and TNNC2 was detected through multiple online databases such as Oncomine, Gene Expression Profiling Interactive Analysis (GEPIA), cBioportal, and UALCAN. Quantitative real-time polymerase chain reaction (qPCR) analysis was adopted for the validation of PYGM and TNNC2 mRNA level in HNSCC tissues and cell lines. Survival curves were plotted to evaluate the association of these two genes with HNSCC prognosis. It was demonstrated that PYGM and TNNC2 were significantly down-regulated in HNSCC and the aberrant expression of PYGM and TNNC2 were correlated with HNSCC prognosis, implying the potential of exploiting them as therapeutic targets for HNSCC treatment or potential biomarkers for diagnosis and prognosis.

## Introduction

Head and neck squamous cell carcinoma (HNSCC) ranks as the sixth most common cancer all over the world and approximately 600000 new cases occur every year [1,2]. Despite the great efforts into the therapeutic advancements, the overall survival (OS) rate of HNSCC has not markedly improved and the overall prognosis still remains poor [3]. One of the possible reasons for the low survival rate and bad prognosis may be delayed diagnosis. It was reported that a majority of HNSCC patients were diagnosed at an advanced stage due to the asymptomatic nature of early stage disease and lack of effective screening modalities [4]. Therefore, reliable and effective means for the early detection of HNSCC and innovative treatment methods for this malignant tumor should be investigated.

For many years, microarrays based on high-throughput platforms served as invaluable and efficient tools for biomedical research by screening significant genetic alterations. Numerous studies have applied microarrays on different cancers to explore the ectopic gene expression to find out essential genes that may play essential roles in the pathological and prognosis of these cancers. However, due to the relatively high expense of microarrays and the limited sample size, the findings sometimes may not be comprehensive and could not be put into clinical application. Since it has increasingly been recognized that through the combinations of the results from different microarrays based on diverse sample origins, the statistical power is increased, the predictive power is more accurate, and the bias of individual studies can be overcome, we decided to make use of a variety of public database to detect relative reliable target genes for HNSCC [5].

The Cancer Genome Atlas (TCGA) and Gene Expression Omnibus (GEO) are two of the most extensive databases where large amounts of data generated from microarrays or high-sequencing technique are collected [6,7]. The original datasets can be retrieved from the database and therefore researchers could select the specific data that meets their study purpose. More importantly, there emerged a variety of online database, such as Oncomine, Gene Expression Profiling Interactive Analysis (GEPIA), UALCAN and c-BioPortal in which researchers have free access to the wide range of datasets and can even perform analysis directly. Therefore, dependent on the wide range of these databases, new studies that made use of the publicly archived gene expression data in various ways and the reuse of these public data can be very powerful [8]. In particular, the rational combination of the datasets has the potential to predict novel gene biomarkers and investigate important genes involved in disease progression so as to develop precision therapies [9]. For example, a study found several significant genes related with ovarian cancer progression and drug resistance by retrieving the data from TCGA, GEO and Oncomine [10]. Also, immune checkpoint HHLA2 was observed to be up-regulated in clear cell renal cell carcinoma and HHLA2 overexpression leads to a remarkable shorter OS and poorer prognosis, implying it could be a potential prognostic biomarker [11].

So far, a large number of genes have been illustrated to be associated with the process and prognosis of HNSCC. The emerging molecular signatures pioneer a promising field where ectopic gene expression may serve as effective biomarker for HNSCC initiation and treatment methods focused on gene regulation may improve the existing therapeutic strategies. Previous studies have suggested some growth factors, tumor suppressor proteins, cell cycle proteins and cytoskeletal proteins could function as potential molecular biomarkers for HNSCC [12]. In the present study, we utilized bioinformatics approaches to identify differentially expressed genes (DEGs) between HNSCC tissues and normal controls from three independent GEO datasets. Then, we selected PYGM and TNCC2, both were not investigated in HNSCC before, to evaluate their relative expression and the potential roles in HNSCC by several online databases. The study aimed to discover novel biomarkers and potential gene therapy targets for HNSCC.

## Materials and methods

### GEO datasets and data processing

The GEO (http://www.ncbi.nlm.nih.gov/geo/) is a public repository for high-throughput microarray and next-generation sequence functional genomic datasets and the raw data, processed data and metadata can all be obtained from the resources [6]. In this study, we downloaded the original CEL data from three datasets (GSE13601, GSE31056, GSE30784) which performed microarray on HNSCC samples and matched normal tissues to identify DEGs. R language was applied to identify the DEGs and the main procedures were illustrated concisely as follows. We utilized the affy package to perform the background correction and data normalization, including conversion of raw data formats, imputation of missing values and background correction. Then, the samples were subjected to differential expression analysis by using the Limma package. *P*<0.01 and |log(FC)| > 2 were set as the threshold and the genes that met the criteria were screened out as DGEs. The intersection of DEGs from three datasets were performed by the VennDiagram package in R language at last.

### Gene Ontology and KEGG pathway analysis

The Database for Annotation, Visualization and Integrated Discovery (DAVID, https://david.ncifcrf.gov/) is an online biological information database for gene functional analysis [13]. Therefore, to predict the possible functions of the overlapping DEGs and their neighboring genes and the potential pathways they may participate in, these genes underwent Gene Ontology (GO) functional and KEGG pathway enrichment analysis in DAVID. *P*<0.05 was considered as statistically significant.

### Oncomine analysis

Oncomine (https://www.oncomine.org/), an online platform providing cancer microarray datasets and data-mining functions, was aimed at facilitating the discovery of significant genes whose expression was closely related with tumor development and progression [14]. Apart from presenting differential expression analyses comparing a variety of cancers with respective normal tissues, it also provides gene expression in different cancer subtypes and an overview of specific gene expression in a wide range of major cancers. Therefore, we explored the mRNA expression of PYGM and TNNC2 in various types of cancer and examined their levels in different HNSCC datasets by using Oncomine database. *P*-value <0.05, fold change of 1.5, and gene ranking of All were defined as the threshold and all the results are summarized in Figure 5.

### GEPIA

GEPIA (http://gepia.cancer-pku.cn/), a web-based tool based on TCGA and GTEx data, could provide fast and interactive functionalities including differential expression analysis, profiling plotting, correlation analysis, patient survival analysis, similar gene detection and dimensionality reduction analysis [15]. In the current study, we overviewed the relative expression of PYGM and TNNC2 in a good number of cancers and |log_2_FC| = 1.5 and *P*-value =0.05 were set as the cutoff. UALCAN analysis, UALCAN (http://ualcan.path.uab.edu/), an easy to use, user-friendly and interactive web-portal for in-depth analyses of gene expression data on cancers from TCGA [16], could be utilized to analyze the relative expression of target genes across tumor and normal samples and relative clinicopathologic parameters. Therefore, we assessed the relationship of PYGM and TNCC2 expression with the grade and stage of the HNSCC samples by UALCAN analysis.

### UCSC Xena browser

UCSC Xena (http://xenabrowser.net/) is an online database that allows users to explore functional genomic datasets for correlations between genomic and/or phenotypic variables. In the present study, we utilized this free online tool to detect whether the level of PYGM and TNNC2 was correlated with the survival of HNSCC patients from TCGA samples. Patients were grouped into relative high expression group and low- or medium-expression group accordingly and *P*<0.05 was considered statistically significant.

### TCGA and c-BioPortal databases

TCGA is a comprehensive and coordinated project designed to discover essential cancer-causing genomic alterations. Based on these information and datasets, it is likely to improve diagnosis methods, treatment standards and prevent cancer ultimately. So far, TCGA has collected large-scale genome sequencing and pathological data analysis of over 30 human tumors, suggesting that it serves as a well-rounded and reliable cancer database [7]. c-BioPortal (www.cbioportal.org) is an online open-access resource where researches have access to explore, visualize and analyze multidimensional cancer genomics data [17]. In this study, c-BioPortal was applied to access HNSCC (TCGA, Provisional) data. The genomic profiles included mutations, putative copy-number alterations from GISTIC, mRNA expression Z-scores and protein expression Z-scores. OS and disease-free survival (DFS) plotter were drawn according to the instructions on c-BioPortal. Furthermore, to achieve a comprehensive understanding of the DEGs selected in the present study, the gene network of DEGs and the neighboring genes was generated for the analysis of the interaction between these genes.

### Tissue specimens

A total of 60 pairs of HNSCC tissues and adjacent non-tumor tissues were collected from patients who received no chemotherapy or radiotherapy between October 2016 and September 2018 at the Department of Oral Maxillofacial-Head and Neck Oncology, Ninth People’s Hospital, Shanghai Jiao Tong University School of Medicine (Shanghai, China). The protocol of the study was approved by the Review Board of the Medical Ethics Committee of the Ninth People’s Hospital, Shanghai Jiao Tong University School of Medicine and all patients provided written informed consent prior to the present study. All the tissue samples were pathologically confirmed as HNSCC and tumor pathological differentiation and clinical stage were defined according to World Health Organization Classification of Tumors and the TNM classification system of the International Union Against Cancer (1988), respectively. OS was defined as the time from start of treatment until death. Follow-up ended in December 2017 or at death.

### Cell culture

SCC-9, SCC-25 and CAL 27 cells were purchased from the American Type Culture Collection (ATCC, U.S.A.), and the human HNSCC cell lines HN4, HN6 and HN30 were kindly provided by the University of Maryland Dental School, U.S.A. SCC-4, SCC-9 and SCC-25 cells were cultured in Dulbecco’s modified Eagle’s medium/nutrient mixture F-12 (GIBCO-BRL, U.S.A.) supplemented with 10% fetal bovine serum (GIBCO-BRL, U.S.A.) and 1% penicillin and streptomycin at 37°C, 5% CO_2_ in a humidified atmosphere while other cells were maintained in DMEM with the same additives. Furthermore, normal oral epithelial cells which were obtained from primary culture were cultured in keratinocyte serum-free medium (GIBCO-BRL, U.S.A.) containing 0.2 ng/ml recombinant epidermal growth factor (Invitrogen, U.S.A.).

### RNA extraction and quantitative real-time polymerase chain reaction

Total RNA of cells and tissues was extracted using TRIzol according to the manufacturer’s protocol (Takara, Japan). Afterward, total RNA (1 μg) was reverse transcribed into cDNA using a PrimeScript™ RT reagent kit (Takara, Japan). The quantitative real-time polymerase chain reaction (qPCR) experiment was carried out on an ABI StepOne real-time PCR system (Life Technologies, U.S.A.) by using a SYBR Premix ExTaq Reagent Kit (Takara, Japan). The detailed reaction conditions were as follows: 95°C for 5 min, 40 cycles of 5 s at 95°C and 30 s at 60°C. All the primers were designed and synthesized by Sangon Biotech (Shanghai) and the detailed sequences for the primers are as follows. PYGM (forward primer: 5′-CCATGCCCTACGATACGCC-3′ and reverse primer: 5′-TAGCCACCGACATTGAAGTCC-3′), TNNC2 (forward primer: 5′-TGGGGACATCAGCGTCAAG-3′ and reverse primer: 5′-CCAAGAACTCCTCGAAGTCGAT-3′), and GAPDH (forward primer: 5′-ACAACTTTGGTATCGTGGAAGG-3′ and reverse primer: 5′-GCCATCACGCCACAGTTTC-3′). GAPDH was used as the internal reference in the present study. The relative expression of target genes was normalized to GAPDH and 2^−ΔΔ*C*^_t_ method was applied to evaluate relative mRNA levels. All the experiments were repeated for three times.

### Statistical analyses

Statistical analysis in the present study was performed using SPSS 21.0 statistical software package. All numerical data were expressed as mean ± standard deviation (SD) from triplicate experiments and the significant differences between two groups were calculated by Student’s two-tailed *t* test. Kaplan–Meier method was applied to generate survival curves with a log-rank test. The associations between gene expression and clinicopathological characteristics of HNSCC patients were analyzed by Mann–Whitney U-test and Kruskal–Wallis test. *P*<0.05 was defined as statistically significant.

## Results

### Identification of overlapping DEGs in the GEO datasets

**Figure 1:**
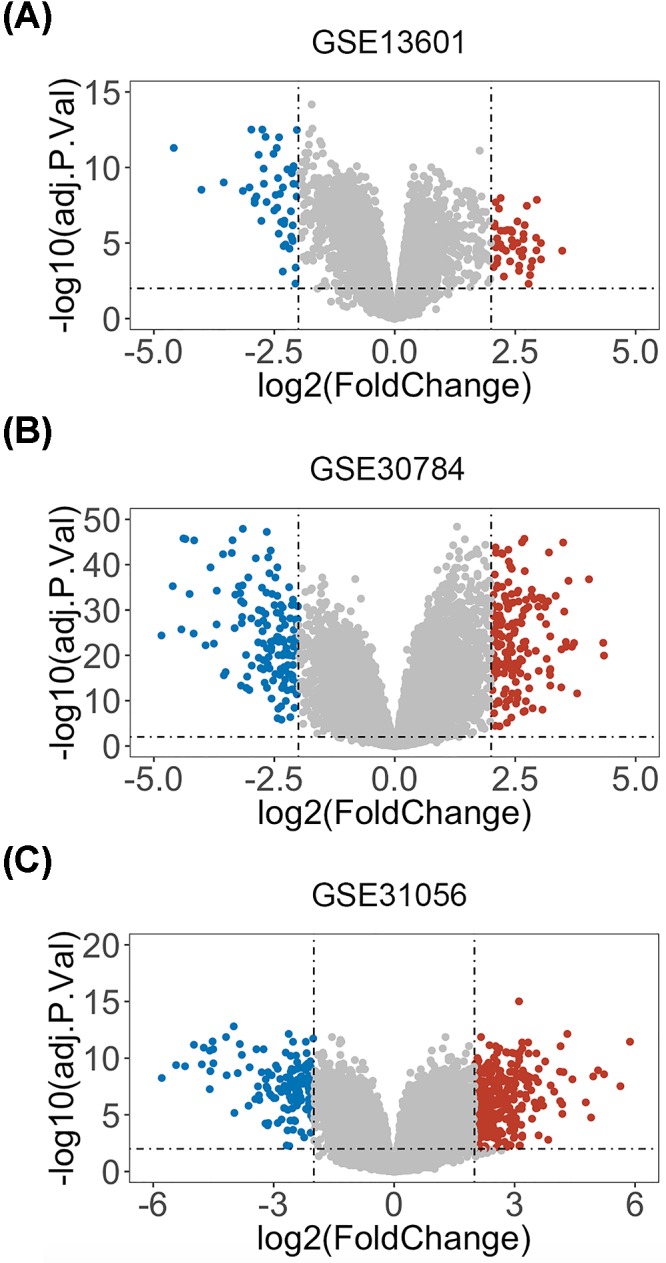
Volcano plot of detectable genome-wide mRNA profiles in three independent GEO datasets (**A**) GSE13601. (**B**) GSE30784. (**C**) GSE31056. Red, blue and gray colors represent relatively high, low and equal expression of genes in the corresponding group, respectively. *P*<0.01 and |log(FC)| > 2 were set as the threshold.

**Figure 2:**
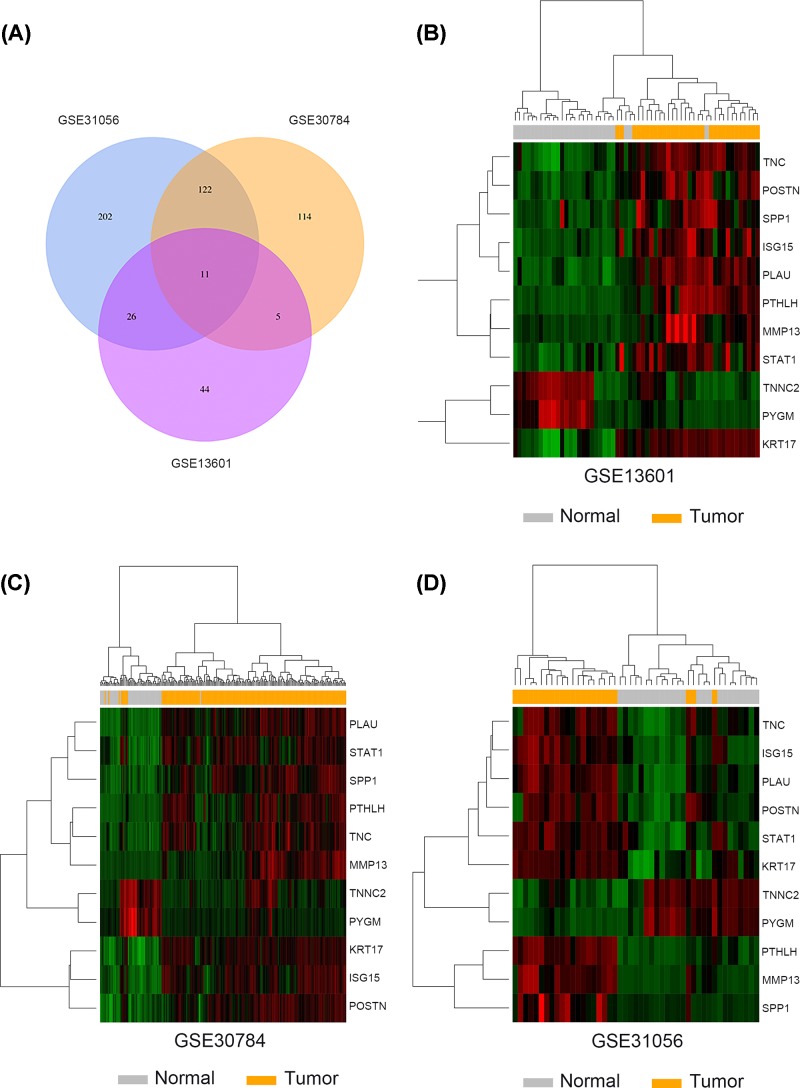
Identification of overlapping DEGs in HNSCC from three GEO datasets (**A**) Venn diagram of overlapping DEGs from intersection of three independent GEO datasets. (**B**–**D**) The heatmaps were drawn to show the overlapping DEGs profiles in HNSCC and normal tissues using microarray data GSE13601, GSE30784 and GSE31056.

A total of three microarray datasets (GSE31056, GSE30784, GSE13601) were obtained from GEO to identify the DEGs between HNSCC tissues and matched normal tissues. The detailed information about GEO datasets are summarized in Table 1. |Log(FC)|>2 and adj.*P*-value <0.01 were defined as threshold. Volcano plots were generated by R language to visualize the distribution of expressed genes between cancer and normal controls from three independent studies (Figure 1A–C). Significantly up-regulated or down-regulated genes were represented by red or green dots, respectively. As suggested by Figure 2A, analysis results of R language manifested 11 genes commonly differentially expressed in these three datasets (*PTHLH, POSTN, SPP1, KRT17, PLAU, MMP13, ISG15, STAT1, TNC, PYGM, TNNC2*). Afterward, we utilized the heatmap2 package in R to generate heatmaps based on the expression levels of common DEGs in GSE31056, GSE30784 and GSE13601 (Figure 2B–D). Each column represented an individual sample and each row represented a specific gene. The color and the intensity indicated the relative expression levels of these DEGs between cancer tissues and normal controls.

**Table 1:** Details of the GEO datasets

Reference	Sample	GEO	Platform	Normal	Tumor
Estilo et al. (2009)	Oral	GSE13601	GPL8300	27	31
Reis et al. (2011)	Oral	GSE31056	GPL10526	24	23
Chen et al. (2008)	Oral	GSE30784	GPL570	45	167

### The network of DEGs and their neighboring genes in HNSCC

**Figure 3:**
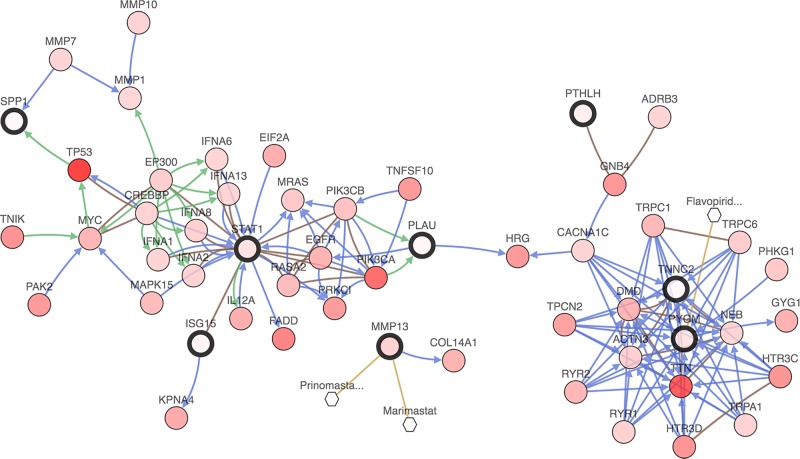
A network of the interaction relationship between overlapping DEGs and the 50 most frequently altered neighboring genes was constructed in c-BioPortal (HNSCC-TCGA, Provisional)

To shed light on the potential mechanism of DEGs involved in HNSCC, a gene regulation network containing DEGs and the 50 most frequently altered neighboring genes was constructed in c-Bioportal. As illustrated by Figure 3, there existed two major modules in the network where STAT1, PYGM and TNNC2 from DEGs played significant roles. A great number of genes were in relationship with these three genes, suggesting the comprehensive and complex functions they played in the biological process of HNSCC. Furthermore, part of the 50 most frequently altered neighboring genes such as *EGFR, PIK3CA* and *TP53*, are well established as essential genes in cancer development and progression. No matter whether DEGs were associated with these important cancer-related genes directly or not, the network implied the possible regulatory role of DEGs in HNSCC. What is more, it was shown that PYGM was related with flavopiridol, an antineoplastic agent by inhibiting most cyclin-dependent kinases (cdks). Previous study has demonstrated that flavopiridol could potently inhibit cell proliferation and induce apoptosis in HNSCC cells [18], suggesting that regulation of PYGM may serve as a promising anti-cancer strategy in HNSCC.

### GO analysis and KEGG analysis of the DEGs and neighboring genes

**Figure 4:**
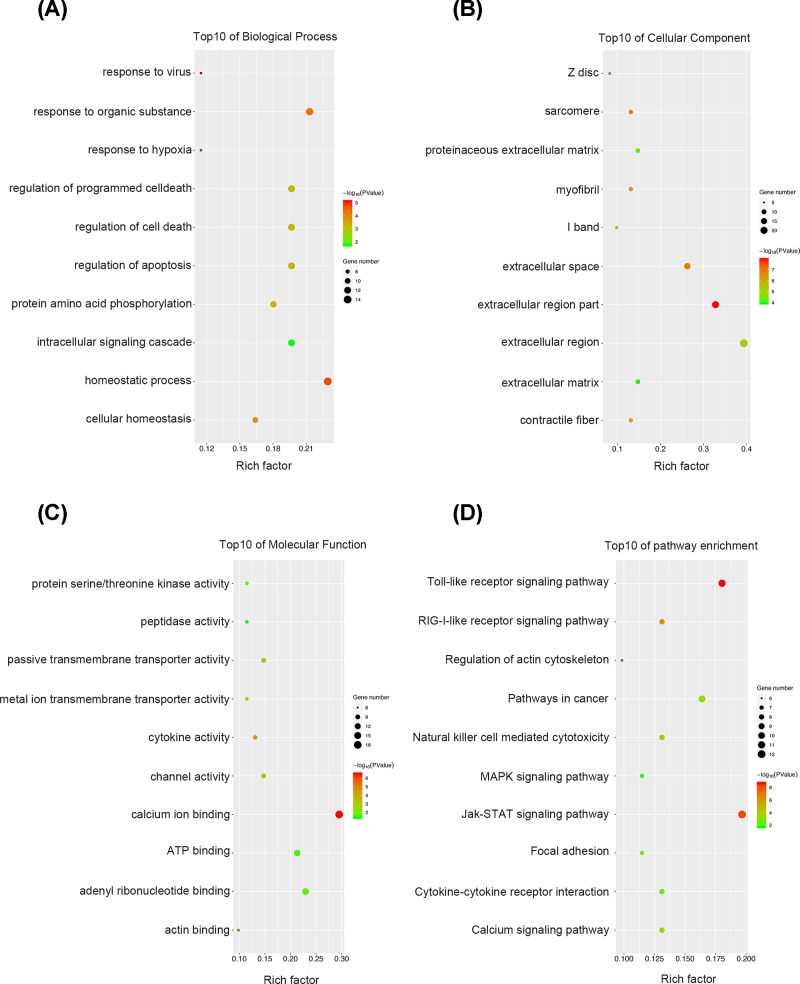
GO analysis and KEGG pathway analysis of the identified DEGs and the 50 most frequently altered neighboring genes in HNSCC (**A**) Top10 of biological process. (**B**) Top10 of cellular component. (**C**) Top10 of molecular function. (**D**) Top10 of KEGG pathway enrichment.

For a more in-depth understanding of the DEGs, GO analyses and KEGG pathway enrichment analyses were performed on the DEGs and the 50 most frequently altered neighboring genes. As illustrated in Figure 4A, for biological process, these genes were mostly associated with homeostatic process, response to virus, hypoxia or organic substance, protein amino acid phosphorylation, regulation of apoptosis and cell death and intracellular signaling cascade. It can be easily concluded that the biological process these genes participated in were closely correlated with cancer development and progression. Regarding cellular component, GO analysis results showed that the DEGs and their neighboring genes were mainly enriched in extracellular region, sarcomere, myofibril and contractile fiber (Figure 4B). For molecular function classification, the genes were significantly enriched in the following functions: calcium ion binding, cytokine and channel activity, transmembrane transporter activity, actin and ATP binding and peptidase activity (Figure 4C). The results from KEGG analysis showed that the among the pathways these genes particularly enriched, many were cancer-related pathways such as Toll-like receptor signaling pathway, JAK-STAT signaling pathway, focal adhesion and MAPK signaling pathway (Figure 4D). The above results indicated that these genes may modulate HNSCC proliferation and metastasis through multiple signaling pathways.

### Overview of PYGM and TNNC2 expression in various cancers and different HNSCC datasets

**Figure 5:**
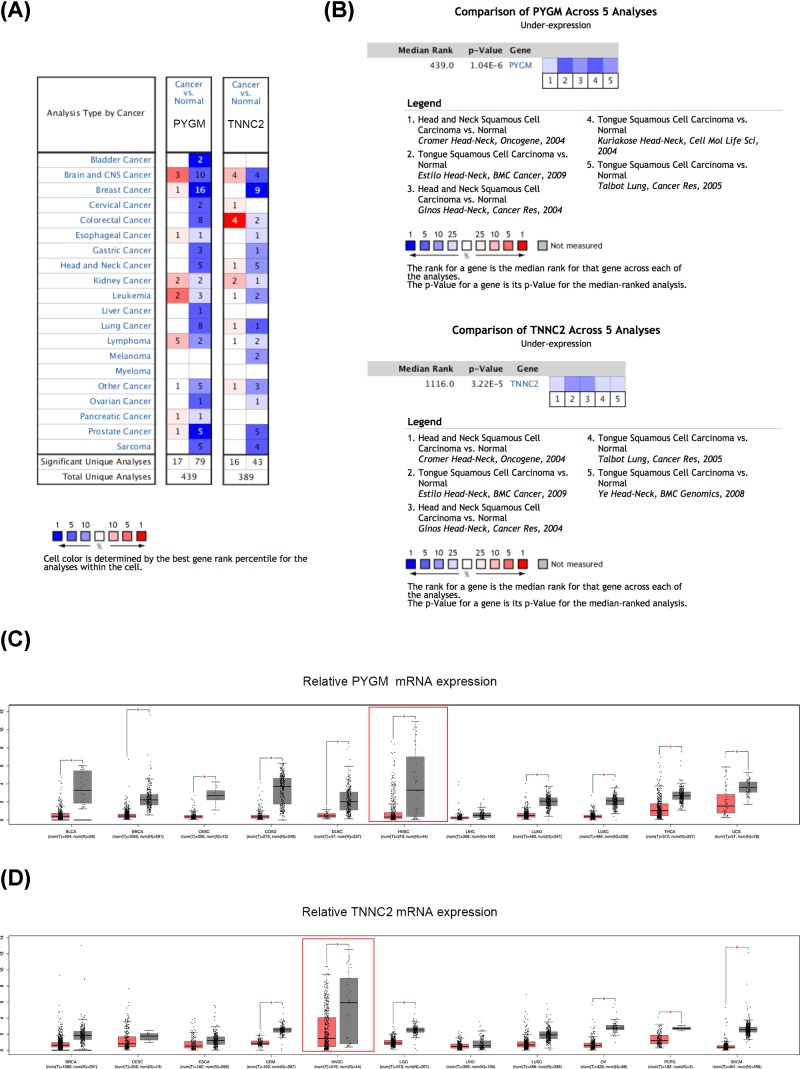
Differential expression analyses of PYGM and TNNC2 in Oncomine and GEPIA (**A**) The transcription levels of PYGM and TNNC2 in different types of cancer in Oncomine. (**B**) Expression analyses of PYGM and TNNC2 in different HNSCC datasets. (**C,D**) An overview of expression of PYGM and TNNC2 in various kinds of cancer in GEPIA. The numbers in colored cells show the quantities of datasets with statistically significant mRNA overexpression (red) or underexpression (blue) of target genes. Cell color was determined by the best gene rank percentile for the analysis within the cells. The threshold was set as gene rank percentile (All), *P*-value (0.05) and fold change (1.5). *, *P*<0.05.

Since we have screened out eleven DEGs from three GEO datasets, we then searched them in the Pubmed to see if they were explored by previous studies. Results manifested that POSTN, SPP1, KRT17, ISG15, STAT1 and TNC were all identified as potential biomarkers in HNSCC by former studies and the relative expression in HNSCC were also further validated by qPCR or IHC experiment [19–23]. Moreover, PTHLH, PLAU and MMP13 were demonstrated to be associated with the survival and prognosis of HNSCC patients and some biological functions of these genes were also validated in *in vitro* experiments [24–26]. However, there are no researches about the relationship of PYGM and TNNC2 with HNSCC and we decided to determine the level of PYGM and TNNC2 in HNSCC and to investigate whether they can act as effective biomarkers for HNSCC. Therefore, we overviewed the transcriptional expression differences of these two genes between cancer tissues and normal tissues in several cancer types and different HNSCC datasets in Oncomine and GEPIA. As shown in Figure 5A–D, mRNA levels of PYGM and TNNC2 were significantly down-regulated in a majority of cancer types and a multiple of independent analyses on HNSCC illustrated the down-regulation of PYGM and TNNC2 in tumor samples. The detailed data about several independent HNSCC datasets in Oncomine are listed in Table 2. From our perspective, the overall underexpression of PYGM and TNNC2 in various cancers may imply the role of them in cancer development and progression and further investigation was of great value.

**Table 2:** The transcription levels of PYGM and TNNC2 between different types of oral cancer and normal tissues (Oncomine)

Types of Cancer vs Normal	Fold change	*t* test	*P*-value	Reference
**PYGM**				
Tongue Squamous Cell Carcinoma vs. Normal	−34.811	−6.544	6.57E-8	Estilo Head-Neck
Tongue Squamous Cell Carcinoma vs. Normal	−1.984	−3.140	0.004	Kuriakose Head-Neck
Tongue Squamous Cell Carcinoma vs. Normal	−5.410	−5.839	1.04E-6	Talbot Lung
Head and Neck Squamous Cell Carcinoma vs. Normal	−14.352	−14.352	−14.352	Ginos Head-Neck
Head and Neck Squamous Cell Carcinoma vs. Normal	−8.701	−3.607	0.017	Cromer Head-Neck
**TNNC2**				
Tongue Squamous Cell Carcinoma vs. Normal	−19.684	−4.452	2.89E-5	Estilo Head-Neck
Head and Neck Squamous Cell Carcinoma vs. Normal	−14.816	−7.846	5.52E-9	Ginos Head-Neck
Tongue Squamous Cell Carcinoma vs. Normal	−9.716	−4.444	3.22E-5	Talbot Lung
Head and Neck Squamous Cell Carcinoma vs. Normal	−13.477	−2.979	0.025	Cromer Head-Neck
Tongue Squamous Cell Carcinoma vs. Normal	−6.408	−2.344	0.017	Ye Head-Neck

### Relationship of PYGM and TNNC2 expression with the clinicopathological parameters of patients with HNSCC

**Figure 6:**
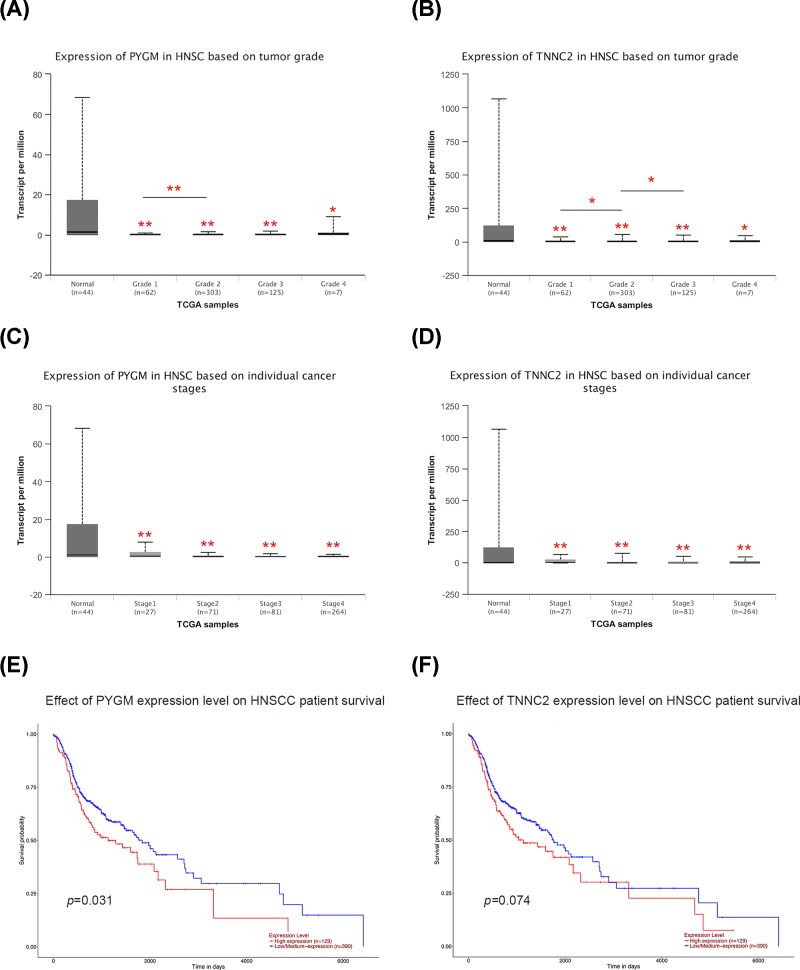
The relationship of PYGM and TNNC2 with clinical parameters and survival of HNSCC patients (**A,B**) The relative expression of PYGM and TNNC2 was correlated with tumor grade in HNSCC. (**C,D**) There was no association between PYGM and TNNC2 with cancer stages in HNSCC. (**E,F**) The prognostic value of PYGM and TNNC2 in HNSCC patients.

Since we have illuminated that the expression level of PYGM and TNNC2 was down-regulated in HNSCC samples, then we focused on whether mRNA expression of these genes was related to cancer grade or stage in individual patients. As shown in Figure 6A, the results indicated that there are significant differences in PYGM expression between patients with Grade 1 and Grade 2. While comparing with HNSCC patients with Grade 1, patients with a more advanced grade (Grade 2 and Grade 3) had a relative lower level of TNNC2 expression (Figure 6B). However, no statistical significance was observed among patients with different stages in both PYGM and TNNC2 expression, implying they may not influence the stage of HNSCC (Figure 6C,D). Taken together, these results indicated that PYGM and TNNC2 may serve as a biomarker for pathological grade in HNSCC and may somehow participate in the progression of HNSCC.

### Prognostic value of PYGM and TNNC2 in HNSCC patients

To further explore the prognostic values of the mRNA expression of PYGM and TNNC2 in HNSCC patients, we conducted survival assay in UCSC Xena database. According to the survival curves in Figure 6E,F, patients with a higher level of PYGM had a better prognosis while the level of TNNC2 seemed to have no statistical influence on the survival time of HNSCC patients. Therefore, the present study suggested that PYGM may be a promising novel predictor for the prognosis of HNSCC.

### Genetic alteration of PYGM and TNNC2 in HNSCC

**Figure 7:**
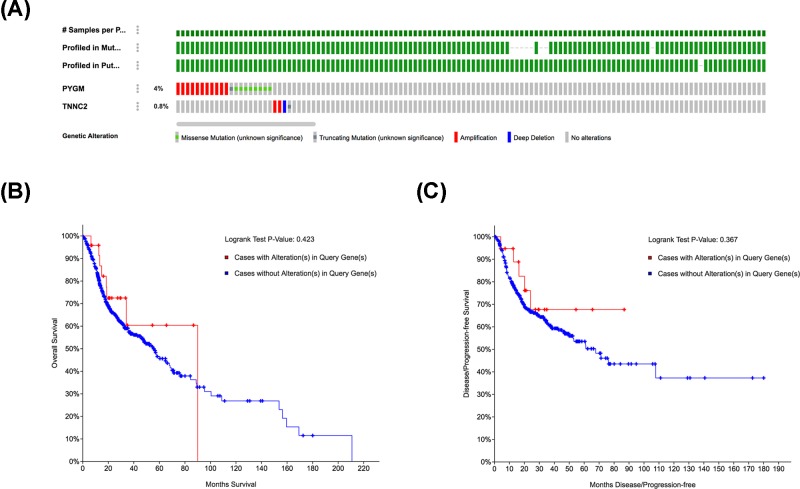
Genetic alteration analysis of PYGM and TNNC2 in HNSCC (c-BioPortal) (**A**) OncoPrint visual summary of alteration on a query of PYGM and TNNC2 in HNSCC. (**B,C**) Kaplan–Meier plots comparing the OS and DFS in HNSCC cases with or without PYGM and TNNC2 gene alterations.

To investigate whether genetic alterations of PYGM and TNNC2 play significant roles in HNSCC, we analyzed the genetic alterations of these two genes by using the cBioPortal online tool. From the OncoPrint schematic, gene alteration of PYGM and TNNC2 was 4 and 0.8% in HNSCC samples, respectively (Figure 7A). These included gene amplification, missense mutation and gene deep deletion. We further evaluated the relationship between gene alteration of these two genes and the survival of HNSCC patients. However, there are no significant differences in OS and DFS in cases with or without alterations in PYGM or TNNC2 (*P*-values, 0.423 and 0.367, respectively) (Figure 7B,C). All the above data suggested that genetic alteration of PYGM and TNNC2 did not play essential roles in HNSCC progression.

### Validation of PYGM and TNNC2 expression in HNSCC cell lines and clinical samples

**Figure 8:**
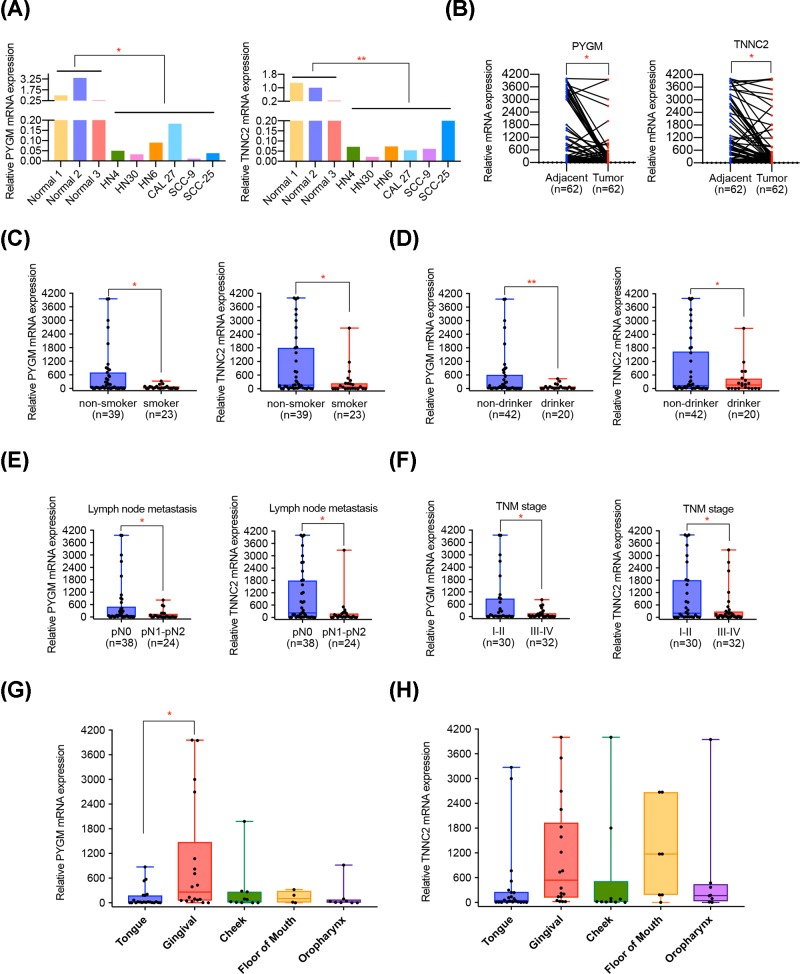
Validation of mRNA level of PYGM and TNNC2 in HNNSCC cell lines and tissues (**A**) The relative expression of PYGM and TNNC2 in HNSCC cell lines and compared normal cells. (**B**) PYGM and TNNC2 mRNA levels were determined by qPCR in 62 pairs of HNSCC tumor samples and adjacent normal tissues. (**C,D**) A significant lower level of PYGM and TNNC2 were observed in HNSCC patients with smoking or drinking history. (**E,F**) The relative lower expression level of PYGM and TNNC2 was correlated with lymph node metastasis and advanced TNM stage in HNSCC patients. (**G,H**) The expression of PYGM and TNNC2 in HNSCC patients with different disease sites.

To further validate the expression level of PYGM and TNNC2 in HNSCC, we performed qPCR experiment in HNSCC cell lines and HNSCC tissues. As suggested by Figure 8A, compared with normal oral cell lines, HNSCC cell lines presented a remarkable lower level of PYGM and TNNC2. Moreover, the results of qPCR conducted on 62 pairs of HNSCC tissues and adjacent matched tissues illustrated that PYGM and TNNC2 were significantly down-regulated in HNSCC tissues (Figure 8B). Interestingly, statistical analysis suggested that PYGM and TNNC2 were both closely associated with smoking and drinking behaviors, with lower levels in patients with smoking or drinking history (Figure 8C,D). We suspected that the components in tobacco or alcohol may influence the expression of PYGM and TNNC2 and further investigation needs to be implemented. What is more, we evaluated the relationship of PYGM and TNNC2 with clinicopathological parameters of patients with HNSCC. It was found that the down-regulation of PYGM and TNNC2 in HNSCC was positively associated with lymph node metastasis and advanced tumor stage (Figure 8E,F; Tables 3 and 4), implying their potential role in HNSCC metastasis and progression. As concluded in Figure 8G,H, most of HNSCC cases came from tongue and gingival and it was suggested that the relative level of PYGM and TNNC2 in patients with gingival cancer were higher than those with tongue tumor. We supposed that the expression of PYGM and TNNC2 may be somehow related with the disease site of HNSCC.

**Table 3:** Relationship between PYGM level and clinicopathologic features (*n*=62)

Characteristics	Number of patients		*PYGM* Δ*C*_t_^1^	*Non-parametric test value*	*P-value*
	Number	%	**Mean** ± **SD**		
**Age (years)**					
<60	24	38.7	194.67 ± 296.46	*Z = −0.159*	*0.874*
≥60	38	61.3	528.18 ± 1081.06		
**Gender**					
Female	19	30.6	643.26 ± 1210.81	*Z = −1.229*	*0.219*
Male	43	69.4	291.19 ± 670.41		
**Smoking history**					
Non-smoker	39	62.9	599.40 ± 1058.01	*Z = −2.149*	*0.032*
Smoker	23	37.1	59.41 ± 80.99		
**Alcohol history**					
Non-drinker	42	67.7	549.89 ± 1032.07	*Z = −2.896*	*0.006*
Drinker	20	32.3	82.38 ± 118.37		
**Tumor size (cm)**					
≤4	42	67.7	533.23 ± 1037.23	*Z = −0.015*	*0.988*
>4	20	32.3	117.38 ± 153.39		
**Lymph node metastasis**					
pN0	38	61.3	575.48 ± 1075.25	*Z = −2.023*	*0.043*
pN1 to pN2	24	38.7	119.79 ± 215.39		
**TNM stage**					
I-II	30	48.4	690.89 ± 1185.17	*Z = −2.040*	*0.041*
III-IV	32	51.6	125.51 ± 198.03		
**Pathological differentiation**					
Well	58	93.6	424.82 ± 901.28	*Z = −1.089*	*0.276*
Moderately/poorly	4	6.4	25.83 ± 28.95		
**Disease site**					
Tongue	22	35.5	127.30 ± 232.55	*H = 2.548*	*0.024*
Gingival	18	29.0	974.89 ± 1395.41		
Cheek	10	16.1	275.36 ± 607.44		
Floor of mouth	4	6.5	132.64 ± 151.43		
Oropharynx	8	12.9	138.77 ± 316.22		
**Recurrence**					
No	54	87.1	404.70 ± 907.83	*Z = −0.189*	*0.850*
Yes	8	12.9	361.16 ± 676.78		

Abbreviations: pN, pathological lymph node status; TNM stage, tumor-lymph node-metastasis stage.

1 Δ*C*_t_ indicates the difference in the cycle number at which a sample’s fluorescent signal passes a given threshold above baseline (*C*_t_) derived from a specific gene compared with that of β-actin in tumor tissues.

**Table 4:** Relationship between TNNC2 level and clinicopathologic features (*n*=62)

Characteristics	Number of patients		TNNC2 Δ*C*_t_^1^	*Non-parametric test value*	*P-value*
	Number	%	Mean ± SD		
**Age (years)**					
<60	24	38.7	749.55 ± 1197.55	*Z = −0.173*	*0.862*
≥60	38	61.3	708.80 ± 1165.07		
**Gender**					
Female	19	30.6	783.31 ± 1177.12	*Z = −0.970*	*0.332*
Male	43	69.4	675.88 ± *1165.07*		
**Smoking history**					
Non-smoker	39	62.9	953.80 ± 1345.13	*Z = −2.033*	*0.042*
Smoker	23	37.1	293.37 ± 592.94		
**Alcohol history**					
Non-drinker	42	67.7	870.62 ± 1323.56	*Z = −2.021*	*0.048*
Drinker	20	32.3	368.99 ± 631.11		
**Tumor size (cm)**					
≤4	42	67.7	902.53 ± 1327.14	*Z = −0.783*	*0.434*
>4	20	32.3	301.97 ± 547.76		
Lymph node metastasis					
pN0	38	61.3	1004.83 ± 1317.18		
pN1 to pN2	24	38.7	240.10 ± 659.55	*Z = −2.211*	*0.027*
**TNM stage**					
I-II	30	48.4	1025.62 ± 1397.33	*Z = −2.098*	*0.041*
III-IV	32	51.6	411.78 ± 810.08		
**Pathological differentiation**					
Well	58	93.6	751.19 ± 1193.18	*Z = −0.917*	*0.359*
Moderately/poorly	4	6.4	94.18 ± 108.96		
**Disease site**					
Tongue	22	35.5	403.26 ± 906.09	*H = 1.943*	*0.051*
Gingival	18	29.0	1097.50 ± 1274.81		
Cheek	10	16.1	605.46 ± 1316.16		
Floor of mouth	4	6.5	1008.61 ± 1223.98		
Oropharynx	8	12.9	708.80 ± 1165.07		
**Recurrence**					
No	54	87.1	641.97 ± 1092.02	*Z = −0.892*	*0.372*
Yes	8	12.9	708.80 ± 1165.07		

Abbreviations: pN, pathological lymph node status; TNM stage, tumor-lymph node-metastasis stage.

1 Δ*C*_t_ indicates the difference in the cycle number at which a sample’s fluorescent signal passes a given threshold above baseline (*C*_t_) derived from a specific gene compared with that of β-actin in tumor tissues.

## Discussion

HNSCC ranks as the sixth most prevalent cancer and constitutes approximately 5% of all malignancies worldwide [27]. The recurrence, metastasis or therapeutic resistance may result in the poor prognosis of HNSCC patients in spite of great improvement on therapy methods. Although there have been numerous studies on a variety of potential biomarkers, the molecular mechanisms underlying the pathogenesis of HNSCC remains elusive [28]. Hence, identifying novel and reliable diagnostic markers and treatment targets is challenging and urgently needed.

HNSCC is characterized by aberrant molecular signatures with various activated oncogenes and inactivated tumor suppressor genes [29]. Recently, a good number of studies have discovered novel genes associated with HNSCC in different ways. It was verified that increased expression of BAG-1 might be a biomarker for cisplatin resistance in patients with primary or recurrent HNSCC [30]. Also, high PD-1/PD-L1 expression was illustrated to be strongly related with radiosensitivity, suggesting that the combination of radiotherapy and anti-PD-1/PD-L1 therapy may improve the prognosis of HNSCC patients [31]. Therefore, the clarification of molecular mechanisms underlying HNSCC development and progression would assist us to discover novel treatment strategy.

Gene expression profile microarrays provide data from different samples and can be utilized for the identification of DEGs by bioinformatics analysis [32]. A multiple of public online databases allowed for data mining and the comprehensive analysis of various databases enhanced the reliability and accuracy of the results. In the present study, we made use of three independent GEO datasets to detect DEGs in HNSCC. Given the GPL platform of these datasets were different (summarized in Table 1), we should analyze original CEL data in R language, respectively. We utilized the affy package to perform the background correction and data normalization, including conversion of raw data formats, imputation of missing values and background correction. Then, the samples were subjected to differential expression analysis by using the Limma package. Through the combinations of the results from different microarrays based on diverse sample origins, the statistical power is increased, the predictive power is more accurate and the bias of individual studies can be overcome. Finally, we screened out eleven DEGs commonly abnormally expressed in three GEO datasets. To trace the origins of gene dysregulation, we constructed an interactive network of eleven DEGs along with the top 50 frequently altered neighboring genes on HNSCC. From the gene network, some important cancer-related genes such as EGFR, TP53 and MYC, could be observed to be correlated with DEGs directly or indirectly. Since these genes have been demonstrated by numerous studies for their significant functions in mediating HNSCC progression and even exploited as potential targets for gene therapy [33–35]. We hypothesized that the gene dysregulation of DEGs might be explained by the abnormal activities of these genes and further studies should be implemented to validate the interaction between DEGs and these neighboring genes in the tumorigenesis of HNSCC. Subsequently, GO analysis conducted on the genes of the network showed that they mainly participate in the cellular homeostasis, regulation of apoptosis and cell death and response to virus or hypoxia, which are all important biological process involved in cancer development [36–38]. Meanwhile, most of the genes were enriched in extracellular region and it was established that the complex interactions between tumor cells and the ECM may play significant roles in tumor metastasis [39]. What is more, KEGG analysis results manifested genes in the network are closely associated with Toll-like receptor signaling pathway, Jak-STAT pathway and MAPK signaling, which were reported to be intimately involved in HNSCC progression [40–42]. KEGG pathways can help us have a better understanding of the HNSCC pathology and provide us with a guide to the potential mechanisms underlying DEGs.

Due to a majority of the DEGs investigated by previous studies, we then selected two genes, *PYGM* and *TNNC2*, for further analyses. Through data mining in a variety of databases, we illustrated that PYGM and TNNC2 were remarkably underexpressed in HNSCC, implying their possible role in cancer progression. Furthermore, the results of the research conducted by Dickinson et al. [43] showed that there was statistically significant difference regarding the protein level of TNNC2 in oral squamous cell carcinoma and healthy tissues. Specifically, the protein level of TNNC2 in oral squamous cell carcinoma samples was 11.5-fold lower than that in normal tissues. It showed that our findings were consistent with this study and suggested that TNNC2 may be exploited as efficient biomarkers for oral squamous cell carcinoma both in mRNA and protein levels. Subsequently, we further validated the relative lower mRNA levels of PYGM and TNNC2 in HNSCC tissues compared with adjacent normal tissues and demonstrated that the down-regulation of PYGM and TNNC2 was positively associated with lymph node metastasis and advanced tumor stage. *PYGM*, a gene involved in glycogen metabolism [44], has been illuminated to be down-regulated in breast cancer and its expression was related with patients survival time [45]. More importantly, previous study suggested that glycogen metabolism may play a central role in cancer progression. It was demonstrated that compared with normal tissues, solid tumor had a higher glycogen content and the correct storage and management of glycogen may be relevant to cancer cell survival [46–49], implying the possible modulatory effect of PYGM in cancer. Also, another study speculated that PYGM may be implicated in gastric cancer via the insulin resistance pathway [50]. However, there existed no findings about TNNC2 with any tumor type and we supposed that further studies are warranted to elucidate its potential functions in HNSCC carcinogenesis.

In conclusion, the present study identified two dysregulated genes, *PYGM* and *TNNC2*, in HNSCC and illustrated that they may serve as effective therapeutic targets and efficient biomarkers for HNSCC. However, there exists some limitations in the present study. The size of HNSCC samples used in our study for the validation of PYGM and TNNC2 is relatively small. Moreover, the biological effect of PYGM and TNNC2 on HNSCC progression and the underlying mechanism have not been investigated. Therefore, more comprehensive studies still need to be conducted in the future.

## Abbreviations

DAVID: Database for Annotation, Visualization and Integrated Discovery
DEG: differentially expressed gene
DFS: disease-free survival
FC: fold change
GEO: Gene Expression Omnibus
GEPIA: Gene Expression Profiling Interactive Analysis
GO: Gene Ontology
HNSCC: head and neck squamous cell carcinoma
KEGG: Kyoto Encyclopedia of Genes and Genomes
OS: overall survival
qPCR: quantitative real-time polymerase chain reaction
SD: standard deviation
TCGA: The Cancer Genome Atlas
